# Anterior Horseshoe Anal Fistula Treated With IFTAK (Interception of Fistulous Track With Application of Ksharasutra) Technique: A Case Study

**DOI:** 10.1155/cris/7226955

**Published:** 2026-06-10

**Authors:** Anil Kumar

**Affiliations:** ^1^ Department of Shalya Tantra, Faculty of Ayurveda, Institute of Medical Sciences, Banaras Hindu University, Varanasi, India, bhu.ac.in

**Keywords:** anterior horseshoe fistula, IFTAK, ksharasutra therapy

## Abstract

Anterior horseshoe anal fistula is a challenging variant of complex anorectal infections. Classical surgeries, including ksharasutra therapy in these cases may result in extensive postsurgical scarring and anal incontinence. On the other hand, interception of fistulous track with application of ksharasutra (IFTAK) technique has emerged as a novel way of managing anal fistulas because of its nature of minimal invasiveness, ability to cut and heal tissues with controlled fibrosis, and continence preservation. Here, we explain a detailed case study of a 40‐year‐old gentleman who presented with a recurrent anterior horseshoe fistula effectively managed with IFTAK. The digital rectal examination (DRE), proctoscopy, methylene blue test, and probing were done to delineate tracts and confirm the diagnosis. Fistula healed completely and showed no recurrence at 8 months of follow‐up. Compared to traditional methods, the technique results in a shorter hospital stay and a shorter treatment duration.

## 1. Introduction

The colorectal surgeons still face therapeutic challenges for managing fistulas especially when complex tracts and subsequent extensions are there [1]. Horseshoe fistulas are routinely found posteriorly, but at times, they may involve the anterior perianal region as well [2]. Anterior horseshoe fistulae are characterized by a primary intersphincteric or transsphincteric tract with bilateral anterior extensions that curve around the anal canal, forming a “horseshoe” configuration [3].

Modern surgical approaches aim to eradicate sepsis while preserving continence. For complex tracts, options include staged drainage, seton placement, LIFT (ligation of intersphincteric fistula tract), advancement flap repair, endorectal flap, fibrin sealants, and novel techniques such as video‐assisted anal fistula treatment (VAAFT) [4]. However, recurrence and incontinence remain concerns when significant sphincter muscle is divided [4].

Ksharasutra therapy, described in traditional medicine, offers a minimally invasive, sphincter‐sparing alternative for complex fistulas [5]. The technique combines controlled chemical cauterization with mechanical cutting by a medicated thread, promoting gradual excision of the tract with simultaneous healing [6]. Conventional Ksharasutra therapy, though offers a better option, but postoperative pain and lengthy duration of treatment are few drawbacks related to it [7]. To overcome these shortcomings, modified ksharasutra therapy, that is, interception of fistulous track with application of ksharasutra (IFTAK) technique, was developed, which offers the same results with less postoperative scar, pain, and shorter duration of treatment [8].

This case is noteworthy as it demonstrates a recurrent anterior horseshoe fistula and its successful management using the IFTAK technique, avoiding extensive surgery. It highlights a sphincter‐preserving, minimally invasive approach with faster healing and reduced morbidity, offering a practical and cost‐effective alternative to conventional surgeries.

## 2. Case Description

A 40‐year‐old male patient with a 9‐month history of intermittent pain and purulent discharge from the anterior perianal region was operated earlier for no results. No history of systemic illness, tuberculosis, inflammatory bowel disease, diabetes, or immunosuppression was reported. Bowel habits were otherwise normal; there was no fecal urgency or incontinence.

**Figure 1:**
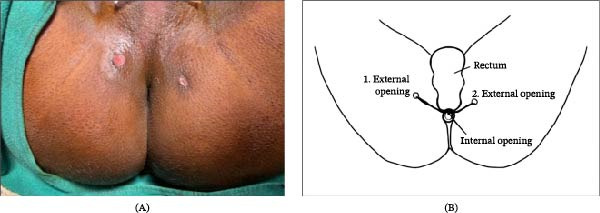
(A) Primary clinical presentation of anal fistula (lithotomy position) with two external openings present at 11 and 2 o’clock positions. (B) Diagrammatic representation of the anal fistula after perrectal examination, internal opening felt anteriorly at 12 o’clock position.

On inspection, two external openings were seen at the 11 and 2 o’clock positions (Figure 1) anterior to the anal verge (patient in lithotomy). Palpation revealed indurated cords tracking subcutaneously. Bi‐digital rectal examination (DRE) was performed using the index finger within the anal canal and thumb externally over the perianal region to palpate the tract between the fingers. This revealed preserved sphincter tone and feeling of a pit anteriorly at the 12 o’clock position; on pressing, pus was oozing from both external openings. Proctoscopy showed mucosal irregularity anteriorly; an internal opening was detected at around 12 o’clock, which was further confirmed by probing and the methylene blue test. After all efforts, it was verified as a recurrent anterior horseshoe fistula. MRI and perianal ultrasonography were not performed as the fistulous tract was adequately identified through clinical examination and intraoperative findings, making additional imaging unnecessary and avoiding extra cost to the patient.

**Figure 2:**
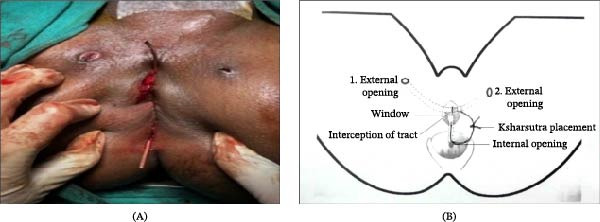
(A and B) Diagrammatic illustration of the IFTAK technique: an anterior window is created, the tracts are intercepted and severed from the proximal tract, and a pliable probe is used to put the ksharasutra in the proximal tract.

In IFTAK operation, the lithotomy posture was applied while the patient was under local anesthesia (2% lignocaine with adrenaline). A vertical incision of about 2 cm was performed at the level of the external sphincter, anterior to the anal canal. The incision was then deepened and widened by blunt dissection to reach the fistula tract. The tract was cut and divided into proximal and distal parts. Then, using a flexible probe, an apamarg ksharasutra [5], a medicated seton prepared by coating a surgical thread with the alkaline extract (Kshara) of *Achyranthes aspera*, known for its cauterizing, debridement, and healing properties, was taken out through the anterior window after being introduced through the internal opening and tied [9] (Figure 2). The good thing about the procedure is that it takes only 15–20 min to complete. After 2 days of primary care, the patient was released with the recommendation to take Tab. *Triphala guggulu*: a classical polyherbal formulation containing triphala (*Emblica officinalis*, *Terminalia chebula*, *Terminalia bellirica*) along with *Commiphora* mukul, known for its anti‐inflammatory, antimicrobial, and wound‐healing effects, 2 BD, daily sitz bath, and *Panchvalkal* cream, a topical preparation derived from the bark of five medicinal plants (commonly *Ficus* species) with astringent and antimicrobial properties, for local application and, 10 g of triphala powder at night [5], which is a traditional herbal combination of the three fruits (*Emblica officinalis*, *Terminalia chebula*, and *Terminalia bellirica*) commonly used for its mild laxative, detoxifying, and gut‐regulating effects, thereby facilitating smooth bowel movements and promoting wound healing.

**Figure 3:**
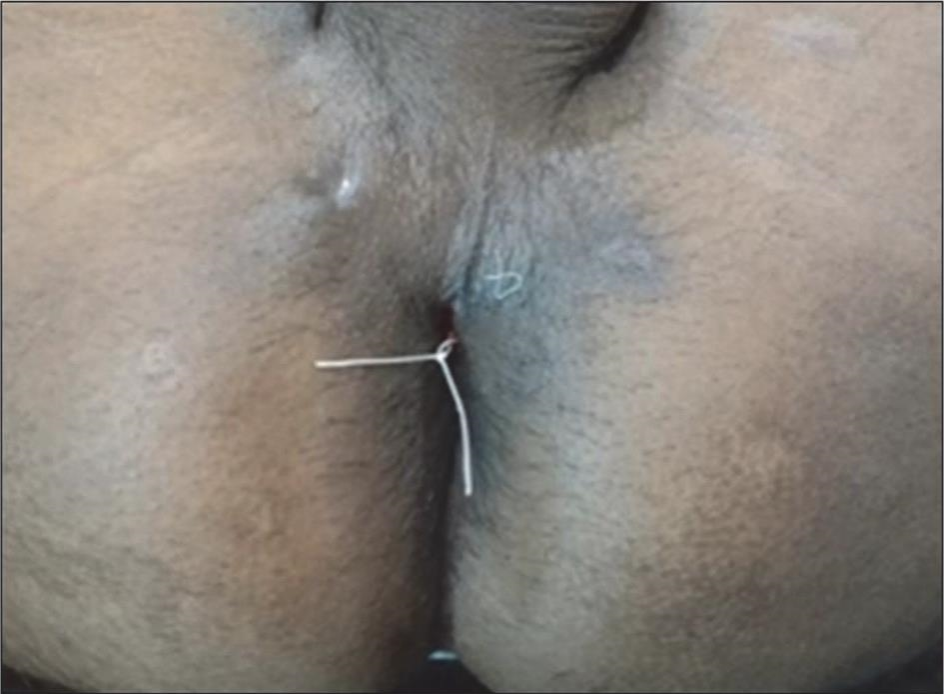
Both external openings healed after 2 weeks of treatment, which shows that the IFTAK was performed accurately.

**Figure 4:**
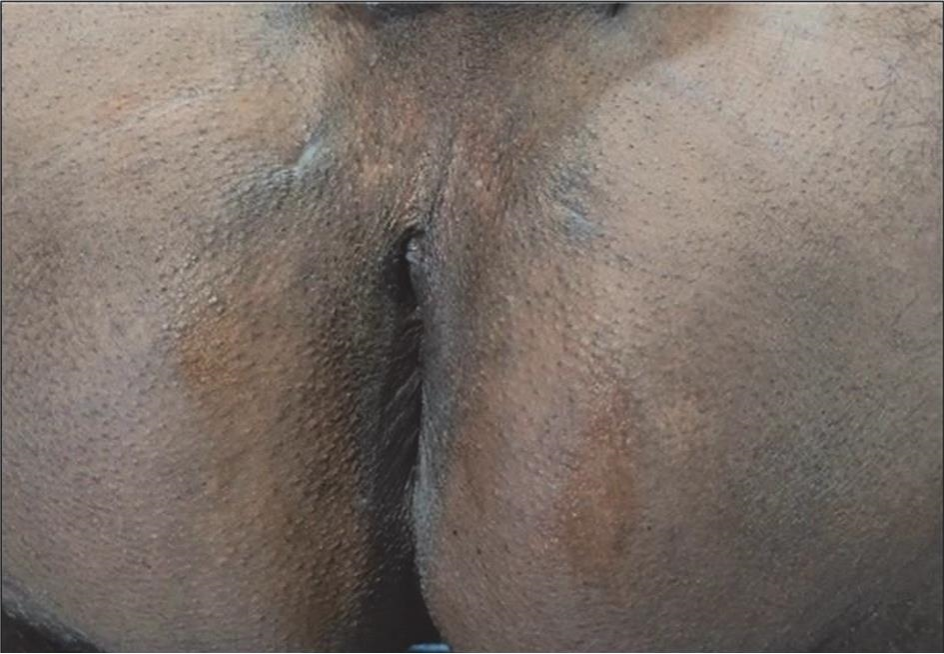
Completely healed fistula after 2 months of treatment.

Ksharasutra (Guggulu based) [10] was changed at weekly intervals. Initially, mild pus discharge persisted from the anterior window, but this gradually subsided. Within 2 weeks, external openings dried (Figure 3). After 2 months, the fistula healed completely (Figure 4). The patient was asymptomatic, fully mobile during the recovery phase, and reported no signs of anal incontinence when questioned as per the Wexner Incontinence Scoring [11]. At 8 months of follow‐up, no recurrence was reported. The sequence of clinical events, interventions, and outcomes is summarized in Table 1.

**Table 1:** The timeline for medical events and treatments.

Time‐line	Medical events
April, 2023	Recurrent boils in perianal regions, consulted local doctors, where he was operated once
January, 2024	After multiple visits when fistula did not heal, patient was referred to National Resource Centre on Ksharasutra Therapy (NRC), Banaras Hindu University (BHU), Varanasi, Uttar Pradesh, India
February, 2024	At NRC OPD, he was thoroughly examined clinically and the case was diagnosed as anterior horseshoe fistula (recurrent)
5 February, 2024	Patient was operated by IFTAK technique
7 February, 2024	After 2 days of primary care, he was released
Ksharasutra was replaced every week	
4 March, 2024 cut through was done 8 April, 2024 fistula completely healed	
At 8 months of follow‐up, no recurrence was reported	

## 3. Discussion

Recent literature highlights significant advancements in the management of complex anal fistulas, with emphasis on minimally invasive and sphincter‐preserving approaches. Techniques such as LIFT, VAAFT, and FiLaC (fistula laser closure) have gained popularity due to their ability to treat fistula tracts with minimal sphincter damage and improved patient comfort [4]. Furthermore, emerging modalities like stem cell therapy have shown promising results, particularly in complex and recurrent fistulas, and are now being considered in recent clinical guidelines as adjunct or second‐line treatments [12].

Conventional surgical methods require prolonged healing and carry the risk of sphincter damage [13]. Although classical Ksharasutra therapy carries strong evidence of its efficacy in fistulas, opting for it for the complex anal fistulas like horseshoe and high‐level type causes postoperative scarring, lengthy treatment duration, and discomfort to the patients [5].

The IFTAK method represents an important modification aimed at addressing these shortcomings. The given technique directly targets the infected anal crypt, which is the primary cause of infection, and it also divides the fistula tract into proximal and distal parts at the level of the external sphincter through interception via an artificially created window. The technique facilitates effective drainage of the pus through the created window and promotes faster healing while reducing tissue injury [4]. The distal tract heals on its own as no infectious material travels to it as it comes out from the window now. In the present case study, a recurrent anterior horseshoe fistula was successfully treated with the IFTAK approach, resulting in complete cure within 2 months and reported no recurrence at the 8‐month follow‐up. The case highlights the safety profile of the procedure as the anal continence was maintained. Previously reported case reports and series of IFTAK have demonstrated complete healing with preservation of continence and low recurrence rates, typically within 2–3 months of treatment [5, 8]. Similar outcomes are seen in this case also, where complete healing occurred within ~2 months with no recurrence, thereby corroborating these findings.

In the present case, the IFTAK technique resulted in satisfactory healing with preservation of continence and allowed an early return to routine activities. However, being a single case report, these findings should be interpreted with caution. It is not appropriate to generalize or infer superiority of the technique, and further well‐designed studies with larger sample sizes are required to establish its clinical efficacy.

## Funding

No funding was received for this manuscript.

## Consent

Prior to surgery and also for publishing the patient’s details, the patient gave his informed consent.

## Conflicts of Interest

The author declares no conflicts of interest.

## Data Availability

The data that support the findings of this study are available from the corresponding author upon reasonable request.
